# A portable and low-cost fluorescence reader for near-patient nucleic acid amplification assays

**DOI:** 10.1007/s10544-026-00801-5

**Published:** 2026-02-14

**Authors:** Ethan Rosenfeld, Kathryn Pacheco, Evan Benke, Ian M. White, Don L. DeVoe

**Affiliations:** 1 Department of Mechanical Engineering, University of Maryland, College Park, MD USA; 2 Fischell Department of Bioengineering, University of Maryland, College Park, MD USA; 3 Fischell Institute for Biomedical Devices, University of Maryland, College Park, MD USA

**Keywords:** Assay automation, Thermal cycler, Microwell arrays, LAMP, Multiplexing

## Abstract

Nucleic acid amplification tests (NAATs) play a critical role in disease diagnostics by enabling rapid and highly sensitive detection of genomic sequences associated with specific pathogens. This paper presents a portable NAAT assay reader combining precise thermal control and spatially-multiplexed fluorescence detection that is designed to extend the applicability of nucleic acid testing across diverse environments. The compact and low cost Multiplexed Array Gene Imager (MAGI) system operates wirelessly, with assay control and readout enabled through a Web-based interface, and can be adapted to a broad range of NAAT formats and assay substrates. Thermal actuation is performed using a printed circuit board heater that provides stable closed-loop temperature control at low manufacturing cost. Performance of the MAGI system is evaluated using a spatially-multiplexed loop-mediated isothermal amplification (LAMP) assay implemented in a custom microfluidic 12-well plate. Overall, the presented system offers a manufacturable, adaptable, and functional platform for use in diverse environments where NAAT cost, portability, and assay flexibility are important considerations.

**Supplementary Information:**

The online version contains supplementary material available at 10.1007/s10544-026-00801-5.

## Introduction

Due to their ability to rapidly detect trace quantities of DNA or RNA, nucleic acid amplification tests (NAATs) have proven indispensable for applications in clinical diagnostics, pathogen tracking, and infectious disease research. Compared with culture- or antigen-based methods, NAATs offer significantly greater diagnostic speed and sensitivity, and can be readily adapted to detect novel pathogens. Despite these advantages, the accessibility of NAATs outside of centralized clinical laboratory settings is limited, with conventional benchtop platforms for nucleic acid amplification and quantification remaining prohibitively expensive, bulky, and complex for emerging applications such as hospital near-patient monitoring, primary care screening, and at-home diagnostics. To bridge this gap and enhance decentralized healthcare delivery, there is a need for novel NAAT platforms capable of improving upon the affordability, portability, and usability of these systems without compromising assay speed, sensitivity, or specificity.

Advances in microfluidic technologies over the past several decades have shown promise in reducing costs and operational complexity for nucleic acid diagnostics (Chen et al. 2022; Li et al. 2022; Mumtaz et al. 2023). However, less attention has been given to the development of platforms capable of automating assay operation for these disposable microfluidic or related microwell devices. In this context, compact systems supporting integrated and quantitative nucleic acid detection are needed to expand the reach of NAAT-based diagnostics (Narasimhan et al. 2023). Due to its high sensitivity, quantitative output, and capabilities for multiplexed analysis, fluorescence detection remains one of the most common sensing methods for assay readout in benchtop nucleic acid amplification platforms, with sensing typically performed using either DNA-intercalating dyes or hydrolysis probes combining reporter and quencher dyes. A number of low-cost and compact fluorescence detection platforms have been reported in recent years that employ LEDs for fluorescence excitation and CMOS cameras for wide-area sensing. For example, several miniature fluorescence microscopes have been developed by adapting conventional fluorescence microscopy optics, consisting of excitation and emission filters with a dichroic beamsplitter, into a smaller form factor suitable for portable imaging applications (Ghosh et al. 2011; Jie et al. 2020; Ma et al. 2020). Other compact fluorescence systems have omitted the use of a dichroic mirror, with excitation and emission filters implemented in both episcopic (Trick et al. 2022) and diascopic (Dong et al. 2023; Kawai et al. 2024) configurations. To further reduce the cost and complexity of the optical system, other fluorescence systems have taken advantage of the relatively monochromatic output of LEDs to avoid the need for an excitation filter, allowing a single emission filter to be used to limit non-specific background fluorescence during sensing (Everitt et al. 2021; Tristan-Landin et al. 2019; Yu et al. 2022).

In addition to wide-field fluorescence detection, portable NAAT platforms also require precise control over the amplification reaction temperature, which must be cycled through multiple set points in the case of polymerase chain reaction (PCR) assays (Bustin 2005) or held at a constant stable value for assays utilizing isothermal amplification methods such as loop-mediated isothermal amplification (LAMP) (Notomi et al. 2015) and rolling circle amplification (RCA) (Ali et al. 2014). A range of portable NAAT assay platforms combining fluorescence detection with either open-loop (Everitt et al. 2021, 2022) or closed-loop (Jie et al. 2020; Maia Chagas et al. 2017; Mendoza-Gallegos et al. 2018; Yu et al. 2022) temperature control have been described, with heating in these systems provided by either resistive (Dong et al. 2023; Everitt et al. 2021, 2022; Jie et al. 2020; Mendoza-Gallegos et al. 2018; Yu et al. 2022) or thermoelectric (Maia Chagas et al. 2017) elements.

A key constraint that can limit the adoption of NAATs in near-patient settings is system cost (Banfi et al. 2024). While the cost for reported portable readers providing both thermal control and fluorescence imaging are significantly lower than commercial benchtop thermal cyclers, which generally cost over $1,000 for systems capable of monitoring a single amplification reaction and over $10,000 for systems designed for analyzing multiple reaction volumes in parallel (Narasimhan et al. 2023), system cost for these miniature readers remains a critical consideration. In the case of emerging portable NAAT platforms, reported component costs are typically on the order of $150–200, in large part due to the use of heating components such as metal ceramic plates (Dong et al. 2023) and thermoelectric elements (Maia Chagas et al. 2017) that represent a large portion of the overall system cost. Heater costs can be reduced by combining a simple resistive element with convective air flow to distribute heat within the sample chamber (Mendoza-Gallegos et al. 2018). New approaches to the development of low-cost elements for precise temperature control in portable NAAT systems are thus needed to address this limitation.

Beyond cost, a number of other considerations are also important for evaluating the capabilities of portable NAAT platforms. ideally, systems should be designed to support flexible operation using a variety of different sample configurations. Just as many conventional benchtop thermal cyclers support the use of multiple microwell plate formats and well densities in a single instrument, the ability to operate assays using different microchannel or microwell arrays configurations with a single portable reader is needed to extend the utility of these systems to a wider range of applications. The ideal platform should also be capable of operating in environments where access to wall power, computing infrastructure, and communications may be limited or inconvenient.

Here we present a platform termed the Multiplexed Array Gene Imager (MAGI) that is developed to address several limitations of established portable assay readers. The capabilities of the system are compared with other reported portable fluorescence NAAT readers in Supplementary Table S1. In its hardware design, the MAGI platform is differentiated from other portable readers through the use of a simple resistive heater patterned on a printed circuit board (PCB) to provide thermal actuation during amplification. This approach allows the overall system cost to be minimized by eliminating the need for thermoelectric or resistive cartridge heaters, which represent the primary cost-driver for many existing portable NAAT platforms. Although other low-cost heaters based on standard resistors (Mendoza-Gallegos et al. 2018) or wire coils (Everitt et al. 2021), these approaches do not allow for direct and uniform delivery of heat to planar sample substrates such as microwell arrays or microfluidics chips. When coupled with integrated electronics enabling closed-loop temperature control, the PCB-based heater provides ± 1% accuracy and ± 0.05% stability over a 66 mm^2^ detection area, making the system highly competitive with other portable NAAT readers but at a cost several orders of magnitude lower than thermoelectric (Trick et al. 2022), metal ceramic (Dong et al. 2023; Yu et al. 2022), or polyimide (Everitt et al. 2022) heaters. The MAGI system also advances the field through its software implementation. Operation of the system is entirely wireless, with power provided by a battery pack, and with assay setup, operation, and readout performed using a Web-based interface on a remote computer through flexible WiFi communications provided through the same single-board computer used for real-time assay control. While wireless transfer of captured images has been previously reported for applications in miniature fluorescence microscopy (Kawai et al. 2024), the portable NAAT platform described here enables the complete assay procedure to be performed with fully wireless control. An adaptable software interface enables straightforward assay configuration, quantitative fluorescence detection, and automated reporting of cycle threshold or time-to-positive (TTP) values supporting diverse user-defined assays to be performed from a remote computer. Furthermore, unlike other platforms that support either single-point detection or capture of fluorescence images for downstream analysis, the MAGI software interface provides user-defined control and real-time monitoring of multiple independent fluorescence detection regions over a large area, allowing the system to be used for wide range of assay substrates and target configurations. Performance of the MAGI system is validated using a spatially-multiplexed LAMP assay with multiple reaction volumes monitored in parallel using a custom microfluidic 12-well plate, yielding a detection limit below 2 DNA copies/µL and time-to-positive values around 30 min.

## Materials and methods

### Optics

The optical subsystem comprises an imaging module (CAM-OV5647, InnoMaker) with a compact 39 mm square footprint utilizing a 5 MP CMOS sensor (OV5647, OmniVision), a 494 nm excitation LED (OSRAM), and glass excitation and emission filters (Rosco). The LED and filters were selected based on the absorbance and emission spectra of EvaGreen intercalating dye but can be substituted for different fluorophores as desired. The optical configuration is episcopic, with the LED held at a 45 degree angle to the sample substrate to minimize direct reflectance of excitation light into the detector. The LED was mounted on a PCB driver board adapted from the literature (Tristan-Landin et al. 2019).

### System control

The camera and all electronics are operated by a Raspberry Pi Zero 2 W single-board computer, which was selected due to its compact size, low cost, and wireless capabilities. In addition to the fluorescence LED, the other actuators in the MAGI include a heater, a micro fan, and two low power LEDs. The heater and fan are used for sample heating while the low power LEDs are used to indicate when the device is on and ready to run an assay. All actuators, except the power LED indicator, are controlled by the Raspberry Pi Zero’s GPIO pins using transistors. Furthermore, the electronics also include two thermistors for temperature sensing; the thermistors were read by the Raspberry Pi Zero using an analog to digital converter. All electronics were connected using a PCB, which interfaced with the controller using a 20 × 2 female pin header. A schematic showing the configuration of all electronics together with the layout of the system PCB is provided in Supplementary Fig. S1. The PCB was designed using KiCAD software.

### Thermal control

A custom PCB containing a double-spiral copper trace provides thermal actuation by Joule heating. The heater was designed using EasyEDA software and manufactured by PCBWay. A piece of 125 μm thick black electrical tape was placed on the surface of the heater to reduce background signal in the optical subsystem due to reflection from the PCB surface. For sensing, one thermistor was embedded in the heater board by inserting the sensor into a small hole drilled through the board and using thermal epoxy to secure the thermistor at the upper surface to ensure direct contact with the base of the sample substrate. A second thermistor was held on the top surface of the sample substrate using a 3D printed cantilever mount integrated into the case. The compliant cantilever is designed to allow for secure contact when using sample substrates of different heights in the range of approximately 4–6 mm. The two point temperature measurement configuration allows for linear interpolation to determine the temperature within the sample reaction wells or chambers.

Closed loop control over sample temperature is performed using a software-defined robust PID controller scheme that was selected due to its ability to operate effectively in the presence of uncertainty about the plant dynamics and the presence of unpredictable disturbances (Dorf 2011; Comstock et al. 2009; Zhou and Doyle 1998). Details of the controller design are provided in Supplementary Note S1, Table S2, and Fig. S2. Thermal performance of the system was evaluated for both steady-state temperature stability and transient response. Steady-state performance was evaluated by holding a sample at a target temperature of 60 ℃ for 45 min to mimic an isothermal amplification process, while transient response was characterized by cycling for 60 min between set points of 94 ℃, 60 ℃, and 72 ℃ for temperatures commonly used in PCR for nucleic acid denaturing, annealing, and extension, respectively. The thermal cycler was programmed to hold each set point for 30 s after reaching within 0.5 ℃ of each setpoint. The temperature distribution across the surface of the heater during actuation was characterized using an infrared camera (Seek Thermal).

### Mechanical casing

The casing of the MAGI was designed in SolidWorks and fabricated using a fused deposition modeling (FDM) printer (Prusa MK4S). All parts were printed using matte black polylactic acid (PLA) filament. The casing components were designed to easily connect to each other, with dual hinges to enable sample loading and access to the internal components for assembly and maintenance, and can be fully assembled in under 2 min.

### LAMP assay

To evaluate MAGI system performance, a LAMP assay was implemented to detect the *nuc* gene associated with *Staphylococcus aureus*. Purified methicillin-resistant *S. aureus* (MRSA) genomic DNA (gDNA) was used as a model sample in these experiments. Each sample included 1X isothermal amplification buffer (B0537S, New England Biolabs), trehalose (15% by weight), 0.6 mM dNTPs (N0447S, New England Biolabs), 1X EvaGreen intercalating dye (Biotium), 0.32 U/uL BST 2.0 DNA polymerase (New England Biolabs), and LAMP Primers for the *nuc* gene (0.2 µM F3, 0.2 µM B3, 1.6 µM FIP, 1.6 µM BIP, and 0.8 µM LoopF) (Zhao et al. 2022). MRSA gDNA (ATCC strain TCH1516) was added to each solution at varying concentrations between 1 × 10^0^ and 1 × 10^2^ copies/µL. A no-template control (NTC) was also prepared without the addition of gDNA.

Samples were tested on the MAGI system using a set of custom microfluidic well arrays fabricated from cyclic olefin polymer (COP) thermoplastic using a CNC milling machine. The microfluidic chips included a base well plate containing a 3 × 4 array of microwells positioned beneath an upper flow channel (Supplementary Fig. S3). The cubic wells with side dimensions of 1.5 mm (3.4 µL volume) were spaced on a 2.7 mm grid. The upper channel, 37 mm long, 9 mm wide at its center, and 1.5 mm tall, served to connect the microwell array with inlet and outlet ports for sample and oil delivery. After CNC milling, the COP substrates were solvent polished by suspending them above a dish containing toluene for 10 min. The base well plates were next exposed to oxygen plasma for 2 min, soaked in 20 mg/mL Pluronic L-121 (Sigma Aldrich) for 30 min, and dried on a hot plate for 30 min. The microwell and channel layers were sealed using vapor-phase solvent bonding by suspending each substrate over a dish of toluene for 10 min before aligning and clamping the layers together by hand.

To prepare samples for testing, each premixed solution of gDNA and LAMP reaction components was deposited in an Eppendorf tube and centrifuged at 1400 rpm for 2 min to degas the sample. Silicon oil degassed for 12 h in vacuum was then added to the Eppendorf tube and the tube was centrifuged at 1400 rpm for another 2 min to remove any air within the boundary between the sample and the oil. The sample was loaded into the COP chips using a syringe pump in withdraw mode at 600 µL/min.

All assays were run for either 60 min or until LAMP reactions in all wells resulted in positive amplification events. The MAGI software interface was used to track the fluorescence curves and calculate the TTP of each well in real time.

## Results and discussion

### Device design and assembly

**Fig. 1:**
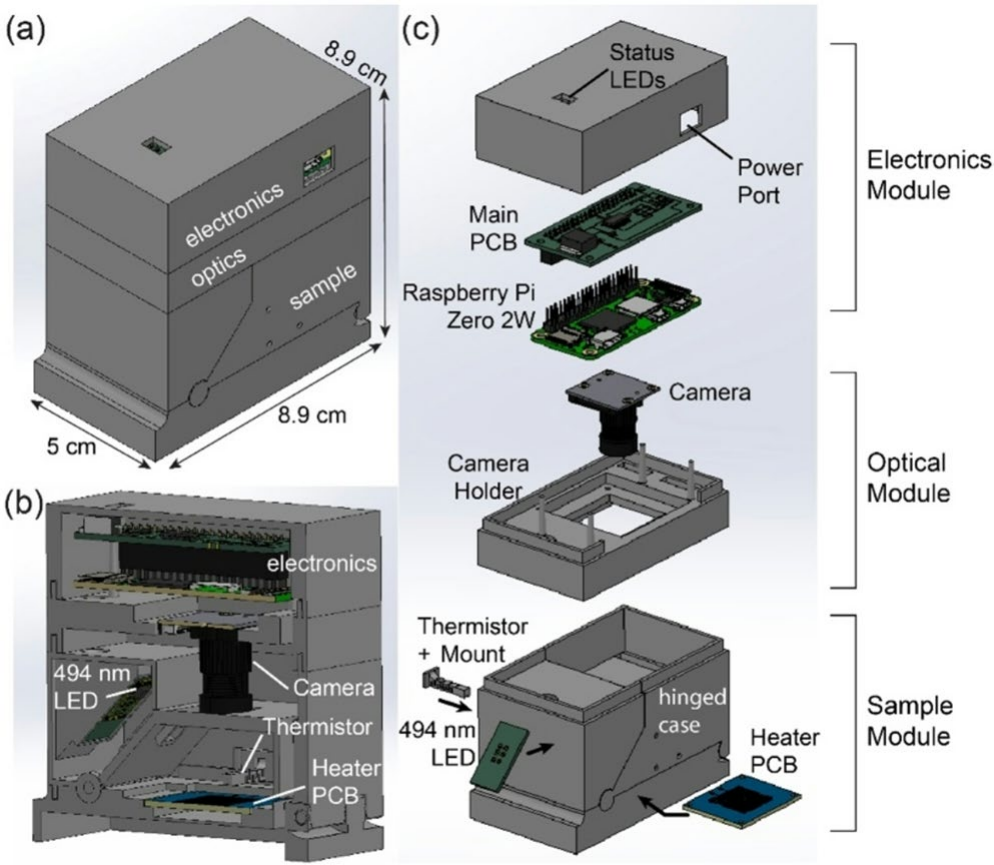
MAGI device overview. (**a**) Overall system dimensions. (**b**) Section view and (**c**) exploded view revealing the internal layout. All casing components were 3D printed using matte black PLA to minimize stray optical signals

**Fig. 2:**
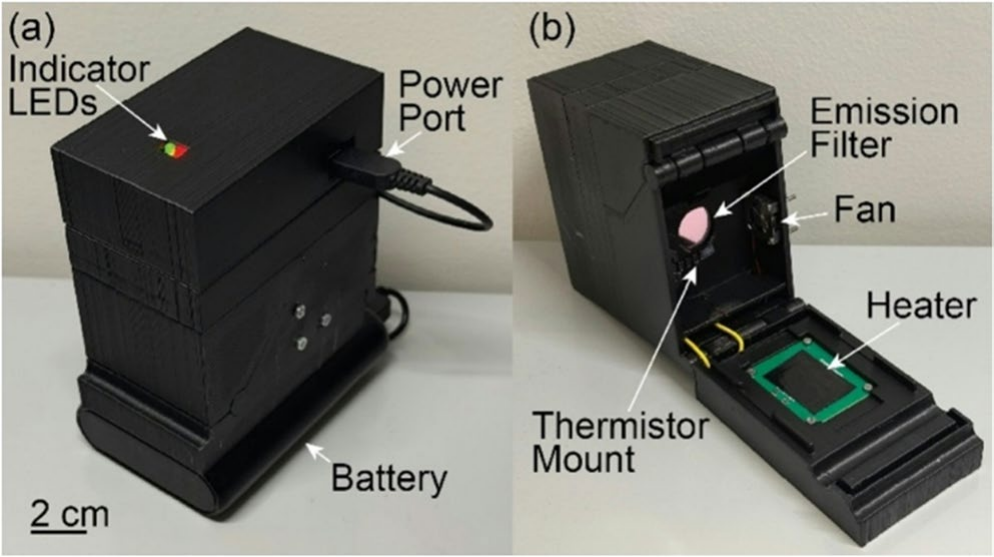
Assembled MAGI system. (**a**) A battery pack powering the device for fully wireless operation and (**b**) the opened device prior to assay card insertion

The MAGI device configuration is presented in Fig. 1. The system consists of 3 integrated modules. A single-board computer (Raspberry Pi Zero 2) and custom daughterboard containing interface electronics for the excitation LED and thermal control system are located in the top electronics module. The daughterboard also contains red and green LEDs to indicate device power and assay status, respectively. The system is powered through the electronics module using either a USB cable connected to wall power or a battery pack for use in situations where access to wall power is limited. The middle optical module contains a compact CMOS camera with wide area lens. The lower sample module holds the excitation LED, optical filters, heater, fan, temperature sensors, and sample holder, with a two-part hinged case that support convenient insertion and removal of different assay substrates. The modular configuration allows for easy device assembly, with 4 separate 3D printed parts making up the mechanical casing. The hinged design allows the closed system to block external light during fluorescence measurements while enabling intuitive access to the sample mount for assay substrate insertion. The device maintains fixed alignment between the sample and all system components, ensuring reliable sealing and avoiding the need for manual alignment. Additional details of the hinged design can be seen in Supplementary Fig. S4. A fully assembled MAGI device is shown in Fig. 2. Sources for all design files and codes required for system construction and operation are provided in Supplementary Note S2.

The device features a small overall size, with a volume of 380 cm^3^ and weight of 190 g. These metrics compare well with other reported portable NAAT readers. While many compact fluorescence imagers with thermal control have been developed for nucleic acid testing in recent years, only a subset of these systems are designed to operate with large area sample substrates suitable for multiplexed analysis, and while several of these platforms offer form factors similar to the MAGI system (Dong et al. 2023; Mendoza-Gallegos et al. 2018; Yu et al. 2022), these systems require the use of external controllers, and in some cases also rely on custom heating elements integrated into the sample substrates themselves. The overall component cost of $82 (Supplementary Table S3) is also well below that of other reported systems offering integrated system control, largely due to the use of a custom PCB heater for thermal actuation.

### User interface

The MAGI system is operated through a Web-based graphical user interface (GUI) written in JavaScript/CSS that connects via Wi-Fi to Python-based HTTP server code running on the same Raspberry Pi Zero used for temperature control and fluorescence imaging. The final code was compiled to standalone MacOS and Windows applications using the open-source Electron JS software framework (OpenJS Foundation) to support operation without a Web browser. Supplementary Fig. S5 presents a view of the GUI during operation of an isothermal assay. The GUI provides access to all device control functions, allowing users to specify regions of interest (ROIs), manually capture fluorescence images, start and end an assay, and view real-time plots of sample temperature and fluorescence intensity within each ROI. Both raw fluorescence images and images containing overlayed ROIs may be displayed on demand. The imager parameters are adjustable through the GUI, with the ability to change the shutter time and gains applied to the image to accommodate a wide range of fluorescence intensities. Following assay completion, the interface allows the fluorescence intensity data to be filtered with user-defined control, automatically determines TTP values, saves raw and processed data as CSV files containing the fluorescence and TTP measurements, and allows the user to reboot or shutdown the MAGI system. The user can also view and clear the server log to assist with troubleshooting and monitoring of system status.

Amplification curve filtering is performed using a Butterworth low-pass filter, with GUI elements that support user-defined control over the filter corner frequency to enable high frequency noise in the fluorescence data to be attenuated. Because some intercalating dyes commonly used in nucleic acid assays are fluorescent at room temperature in the absence of DNA, the interface also allows the user to define a threshold time before which the fluorescence data is ignored when calculating TTP results. Similarly, to account for measurement noise at low signal levels, a fluorescence intensity threshold can be set to define a minimum value for fluorescence quantitation. The use of these threshold values allows for effective normalization of all fluorescence data over a [0,1] range for accurate determination of TTP values for each well. The TTP values are automatically calculated by determining the slope at the midpoint of each fluorescence curve and projecting a line from the midpoint back to the time axis with the same slope.

**Fig. 3:**
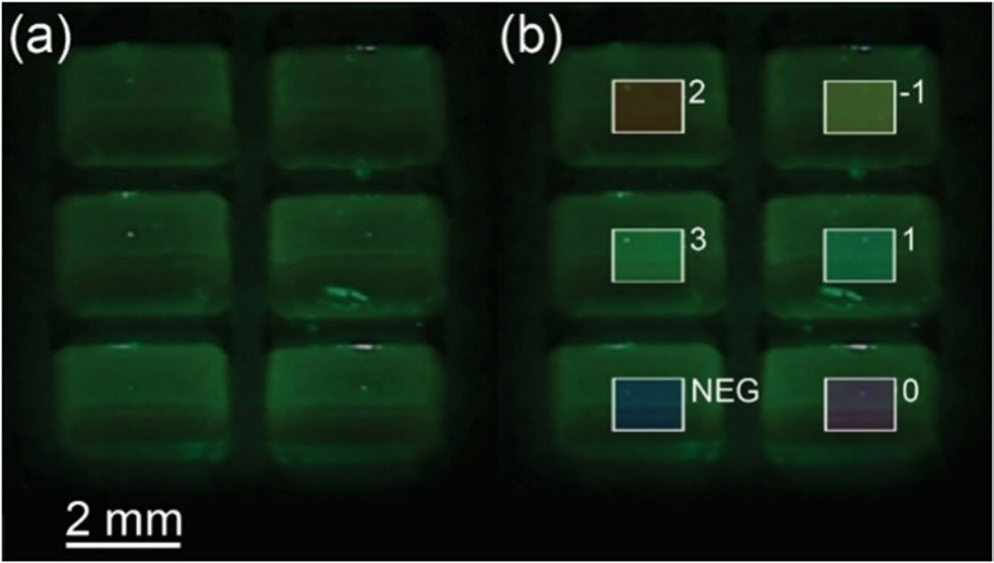
(**a**) Fluorescence images from a 3 × 2 well chip containing EvaGreen intercalating dye at room temperature. (**b**) Identical image with overlay of user-defined ROIs differentiated by distinct labels and corresponding colors

The MAGI system can be easily configured for different assays through an editable text file written in JSON format termed an assay card. The customized assay cards allow the user to define an array of rectangular reaction volumes with details including width and height dimensions for each ROI, coordinates of the upper left corner of the array, and spacing between each array row and column. The assay card format also allows the user to define the locations for different gene targets across the array. In addition, multiple sets of positive and negative amplification results associated with each gene target can optionally be included, with each entry corresponding to the assay output required for positive detection of a specific biological target that may be present in the sample. Example fluorescence images extracted from the MAGI interface are shown in Fig. 3. User-defined ROI names are automatically overlaid on each region with color coding to simplify image interpretation.

### Temperature control

**Fig. 4:**
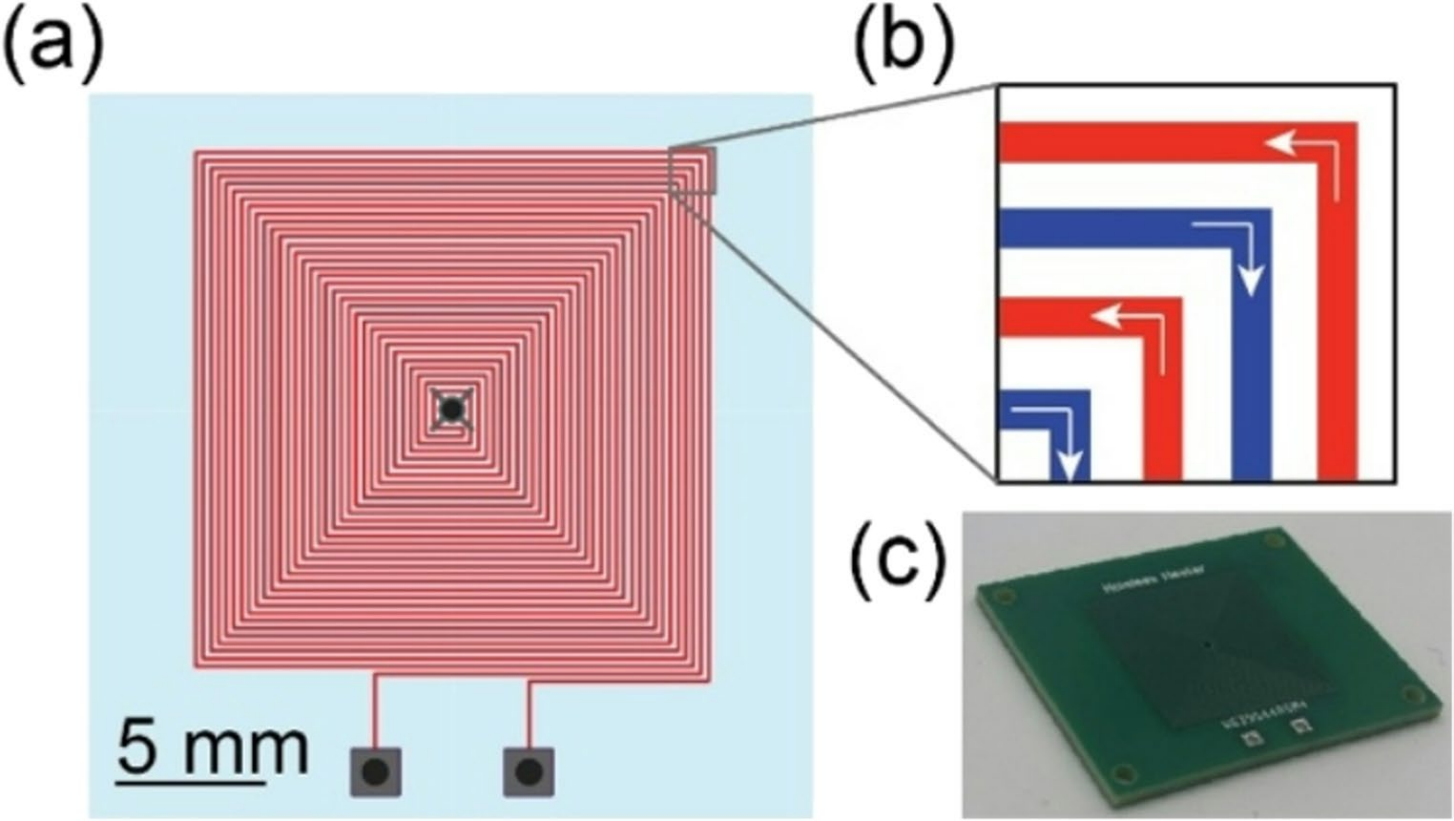
Single-board PCB heater design. (**a**) The double-spiral copper trace has a length of 120 cm, width of 125 μm, and thickness of 35 μm. A hole in the center of the spiral serves as a thermistor mount for temperature measurements of the heater surface. (**b**) Zoomed-in section view showing the direction of current in the double-spiral trace. (**c**) Image of a fabricated PCB heater

Control over sample temperature is achieved using a resistive heater fabricated using copper traces of a PCB and a pair of thermistors positioned on either side of the sample substrate. The lower thermistor is located within a hole drilled in the center of the heater PCB, and the upper thermistor is attached to a flexible cantilever integrated into the sample module case. The cantilever was designed with an accordion geometry to increase compliance, thereby allowing the mount to flex when bringing the thermistor into contact with the upper surface of the sample substrate upon closing the hinged case. The geometry of the sample holder and cantilever support the use of sample substrates with thicknesses in the range of 4–6 mm. Each thermistor is used in a Wheatstone bridge circuit with a 10-bit ADC for digital readout. The dual-thermistor configuration enables monitoring of both the upper and lower sample substrate temperatures, allowing for temperature within the substrate to be estimated by linear interpolation. Details of the PCB heater element are shown in Fig. 4. The heater board employs a square double-spiral coil trace with copper layer thickness of 35 μm, width of 125 μm, and total length of 120 cm, resulting in 4.6 Ω resistance at room temperature. Unlike commercial Peltier (Maia Chagas et al. 2017) or ceramic (Dong et al. 2023) heating elements used in other portable NAAT devices, the cost of the PCB-based heater is similar to that of off-the-shelf power resistors (Mendoza-Gallegos et al. 2018) but offering significantly greater control over spatial heat generation and temperature distribution.

**Fig. 5:**
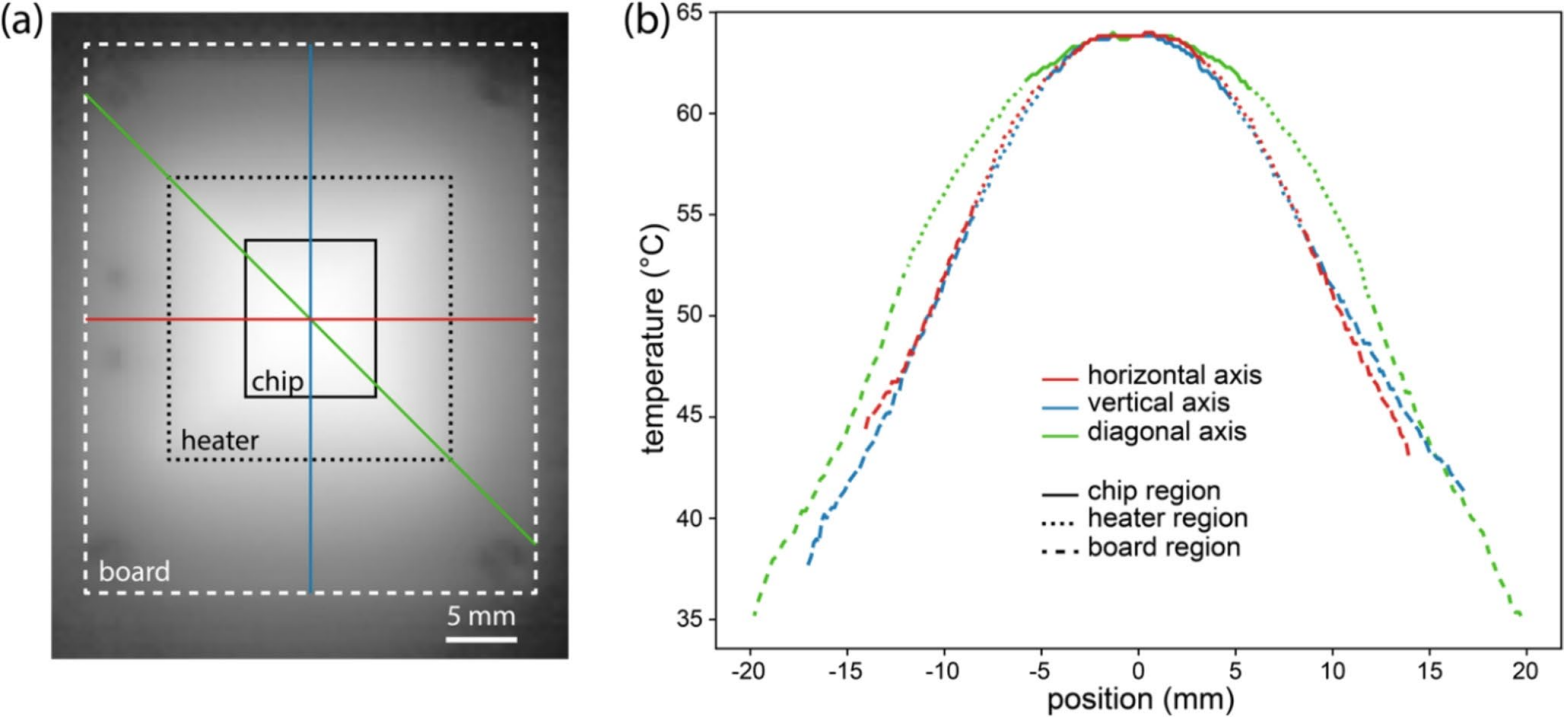
(**a**) Thermal image of the PCB heater board, with boundaries of the board and heater traces labeled together with the boundary of the microfluidic well array. (**b**) Temperature profiles along the horizontal, vertical, and diagonal axes denoted in panel (**a**)

An IR image of the PCB heater during operation with a 63 ℃ target temperature is shown in Fig. 5a. Regions corresponding to the overall PCB board, the spiral heater trace, and the position of the microwell array used for assay characterization are shown in this figure. Measured temperature profiles along the vertical, horizontal, and diagonal axes across all regions are presented in Fig. 5b. While the horizontal and vertical axes exhibit similar thermal gradients, the temperature along the diagonal is slightly elevated at a comparable distance from the center of the PCB, likely due to the higher sheet resistance of the traces at each 90 degree turn resulting in a local increase in Joule heating at these positions. Overall temperature variations remain below ± 1% within the microwell array area, and below ± 5% over the full heater area. Because temperature sensing is performed using a pair of thermistors contacting the microwell array chip at specific locations on the top and bottom of the array chip, assay temperature setpoints can be adjusted to calibrate the desired assay temperature based on the measured temperature distribution. The addition of a heat spreader between the PCB and assay substrate may be desirable for applications requiring lower temperature variations over larger areas, at the expense of increased thermal mass. Other design modifications with the potential to further improve heater performance include adopting a circular electrode geometry to eliminate the localized Joule heating observed in the current square design and varying the spiral trace density as a function of radial distance by increasing density toward the outer edges to promote a more uniform temperature distribution.

**Fig. 6:**
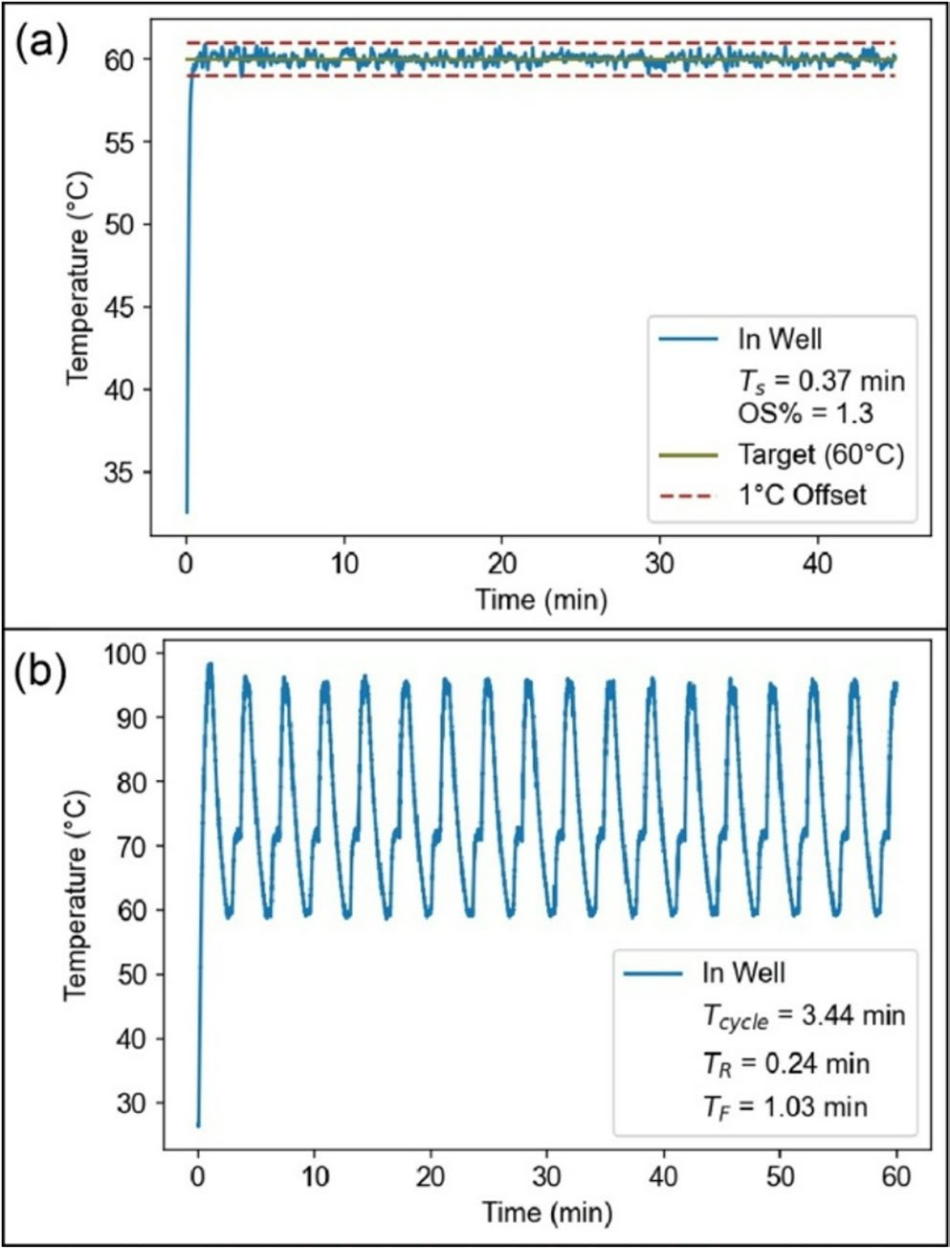
Closed loop in-well temperature response of the MAGI’s thermal control system for (**a**) isothermal control with an average output of 60.03 ℃ and a standard deviation of 0.29 ℃. (**b**) The PCR thermal response shows a minimum cycle time of 3.44 min

The steady-state temperature response of the thermal control system is shown in Fig. 6a when using the integrated thermistors for two-point temperature sensing. As outlined in Supplementary Note S1, the controller tuning process is straightforward, using the provided scripts (Supplementary Note S2) to estimate the plant transfer function from experimental data and derive the corresponding PID gains. This tuning process should be repeated when employing sample substrates with different dimensions or thermal properties. Following tuning, the system exhibited a measured rise time of 0.20 min, settling time of 0.37 min, and 1.3% overshoot. The steady state error and relative standard deviation of steady state temperature fluctuations were both found to be less than 0.05% of the temperature set point.

Temperature response of the system during thermal cycling is presented in Fig. 6b. When using the same PID controller parameters employed during isothermal testing and holding each temperature setpoint for 30 s, a minimum cycle time of 3.44 min was achieved. As seen in the figure, the cycle time is dominated by a measured fall time of 1.03 min. To reduce thermal cycling times for applications requiring more rapid temperature changes, forced convection within the system may be enhanced by utilizing a larger box fan to increase heat removal efficiency.

### LAMP assay characterization

**Fig. 7:**
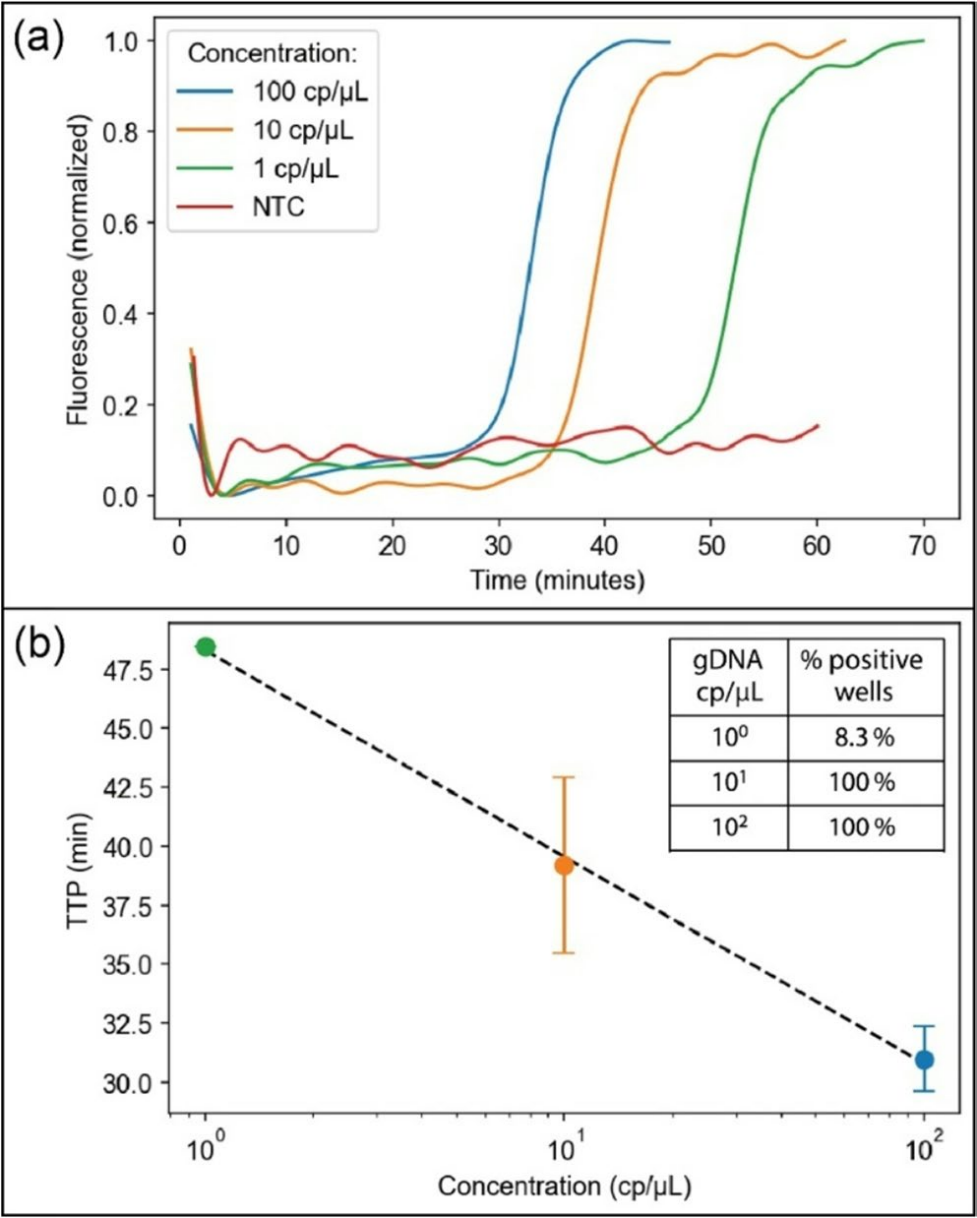
(**a**) Normalized fluorescence amplification curves at varying MRSA gDNA concentrations, and (**b**) extracted TTP values for each concentration (*n* = 12). The percentage of wells exhibiting successful amplification is shown inset

Performance of the MAGI system was evaluated for isothermal amplification of target DNA sequences using a set of thermoplastic microfluidic chips, each containing an array of 12 discrete microliter-scale wells as LAMP reaction chambers. Because the LED used for fluorescence excitation is oriented at 45° to the sample substrate surface to reduce direct reflection of the incident light into the collection optics, the well-based chip design leads to shadowing that results in reduced illumination with a portion of each well volume. This effect can be seen in the fluorescence images in Fig. 3a, where the lower regions of the wells exhibit a reduction in fluorescence emission. To minimize the impact of shadowing on LAMP assay performance, ROIs were selected to define rectangular boxes encompassing the upper half of each well. After validating optical performance using this ROI configuration, a dilution study was performed using varying concentrations of MRSA gDNA to assess linearity and detection limit of the MAGI system for quantifying the *nuc* gene of *S. aureus*. Assays were performed at each target concentration using individual microwell chips, with independent measurements performed in each well using the automated MAGI software (12 measurements per chip). Representative amplification curves at each gDNA concentration level are presented in Fig. 7a. The data in this plot was extracted directly from the CSV data files saved by the MAGI software for each assay run. No amplification was observed in any of the no-template negative control wells lacking gDNA, confirming that the assay is robust against false positives. Extracted TTP values for each sample concentration are presented in Fig. 7b. An inverse log-linear relationship between sample concentration and TTP is apparent in this plot. At sample concentrations of 1 × 10^1^ and 1 × 10^2^ cp/µL, amplification was observed in all 12 replicates, while only one of the 12 wells amplified for the 1 × 10^0^ cp/µL sample. Based on the well volume of 3.4 µL, an average of 3.4 copies of MRSA gDNA per well were present at this sample concentration. As outlined in Supplementary Note S3, given that only 8.3% of the wells in this experiment exhibited amplification, an estimated 6 copies of MRSA gDNA were sequestered in the positive well, corresponding to a limit of detection (LoD) of 1.8 cp/µL. While this estimate is comparable to other LoD values reported for related LAMP assays performed using conventional benchtop thermal cyclers (Dong et al. 2022; Luo et al. 2022), validation of this result will require further experiments employing replicate no-template control samples to determine background signal limits, as commonly used for establishing LoD values in nucleic acid amplification tests (Forootan et al. 2017).

While purified gDNA was used to evaluate performance of the MAGI platform through the model LAMP assay, sample preparation steps such as bulk purification and cell lysis followed by nucleic-acid extraction, isolation, and concentration are often required for characterization of real-world samples. An advantage of LAMP assays is that the *Bst* polymerase used for isothermal amplification is highly tolerant of inhibitors that would degrade typical PCR reactions, significantly reducing the requirements for sample cleanup and allowing the use of sample matrices including saliva, blood, urine, or swabs with rapid thermal lysis serving as the only sample preparation step needed to release DNA from the raw specimens before amplification (Adedokun et al. 2024; Paul et al. 2020; Wilkinson et al. 2024). Although the MAGI system is designed to be assay-agnostic, enabling programmable real-time fluorescence readout for any suitable amplification process, it may find greatest utility for isothermal assays where no sample preparation steps beyond in situ thermal lysis are required, allowing the system to operate in near-patient or point-of-care settings directly from unprocessed clinical specimens.

## Conclusion

The MAGI system is a compact, portable, and low cost fluorescence imaging platform that enables flexible and programmable assays using a wide range of sample substrates. The system employs low cost components and a modular design that leverages 3D printed elements to enable straightforward manufacture and assembly. The use of a robust PID control loop enables a high level of temperature stability, and assay readout is performed with wireless communication through a platform-independent interface that offers a high level of control over key aspects of assay setup and operation. Using an isothermal nucleic acid assay for the detection of a single gene target from MRSA gDNA, the system was shown to enable rapid assays, with time-to-positive values as low as 30 min achieved, and with an estimated limit of detection below 2 cp/µL. Overall, the system provides a turnkey platform that can be easily adapted to diverse applications in nucleic acid testing or other related fluorescence-based assays that require precise thermal control, offering new opportunities for near-patient and point-of-care testing in a wide range of clinical settings where improved access to nucleic acid-based assays is needed.

## Supplementary Information

Below is the link to the electronic supplementary material.

ESM 1 (PDF 3.91 MB)

## Data Availability

No datasets were generated or analysed during the current study.
