# Supplementary material for: A portable and low-cost fluorescence reader for near-patient nucleic acid amplification assays
Source: Biomed Microdevices. 2026 Feb 14;28(1):14. doi: 10.1007/s10544-026-00801-5 (PMC12904924; doi:10.1007/s10544-026-00801-5)
Supplement: Supplementary file 1 — (PDF 3.91 MB) [file 10544_2026_801_MOESM1_ESM.pdf]

# Supplementary Information

# A Portable and Low-Cost Fluorescence Reader for Near-Patient Nucleic Acid Amplification Assays

Ethan Rosenfeld,[1,3] Kathryn Pacheco,[1,3] Evan Benke,[2,3] Ian M. White,[2,3] Don L. DeVoe*[1,2,3]

[1]Department of Mechanical Engineering, University of Maryland, College Park, MD, USA
[2]Fischell Department of Bioengineering, University of Maryland, College Park, MD, USA
[3]Fischell Institute for Biomedical Devices, University of Maryland, College Park, MD, USA

## Contents

Figures:

Figure S1: MAGI electronics system schematic and board layout.

Figure S2: Block diagram of the robust PID system for thermal control.

Figure S3: Microwell chip design used for MAGI system evaluation.

Figure S4: MAGI case design.

Figure S5: Overview of the MAGI graphical interface.

Tables:

Table S1: Performance metrics for portable fluorescence-based nucleic acid amplification platforms.

Table S2: Transfer functions and prefilter parameters for the thermal control system.

Table S3: MAGI system bill of materials.

Notes:

Note S1: Thermal control system design.

Note S2: Links to design file and code repositories.

Note S3: Calculation of detection limit.

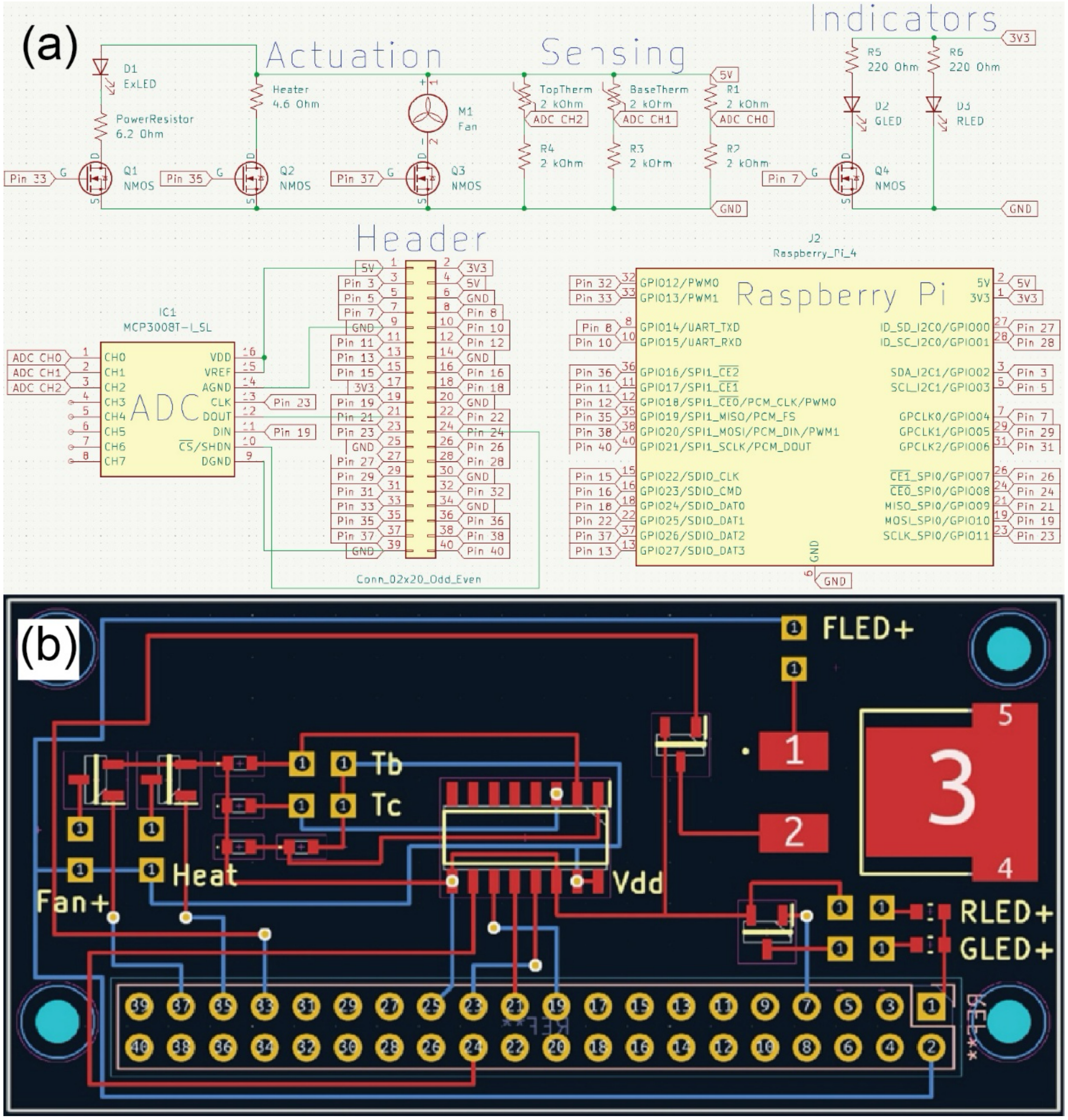


**Figure S1: MAGI electronics system schematic and board layout.** (A) Schematic of the electronics subsystem. The Raspberry Pi Zero 2 W interfaces with a 2 x 20 female header connected to the system's PCB. The actuation subsystem includes the excitation LED, heater, and fan. The sensing subsystem comprises the three branches of the Wheatstone bridge for temperature measurement, with one branch for the upper thermistor, one branch for the base thermistor, and a shared reference branch. An ADC is used to read the output voltage of each branch. The indicator subsystem includes red and green LEDs used to report system power and assay operation status, respectively, to the user. Matching labels in the schematic are used to indicate connected nodes to ensure clarity in the schematic. (B) Layout of the system PCB housing all components shown in panel (A).

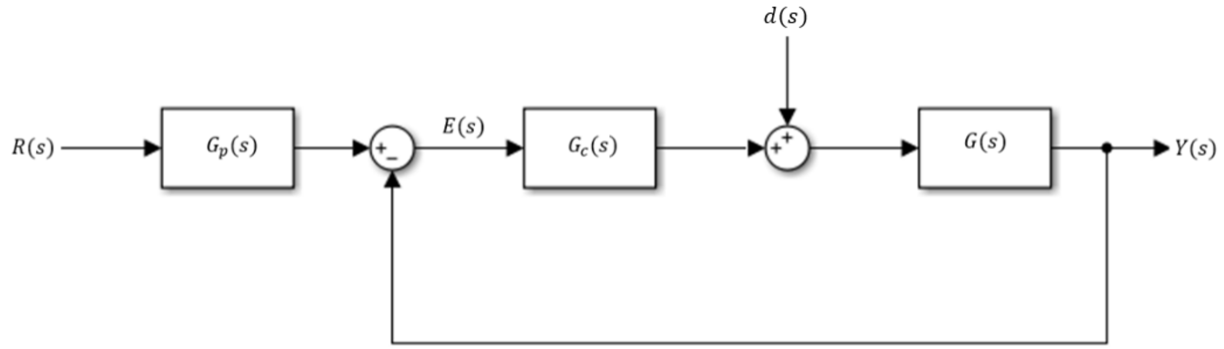


**Figure S2: Block diagram of the robust PID system for thermal control.** The control loop including a reference temperature R(s), prefilter $G_p(s)$ to condition the reference, PID controller $G_c(s)$, MAGI thermal system plant $G(s)$, output temperature $Y(s)$, error between the output and the conditioned reference $E(s)$, and disturbance signal $d(s)$.

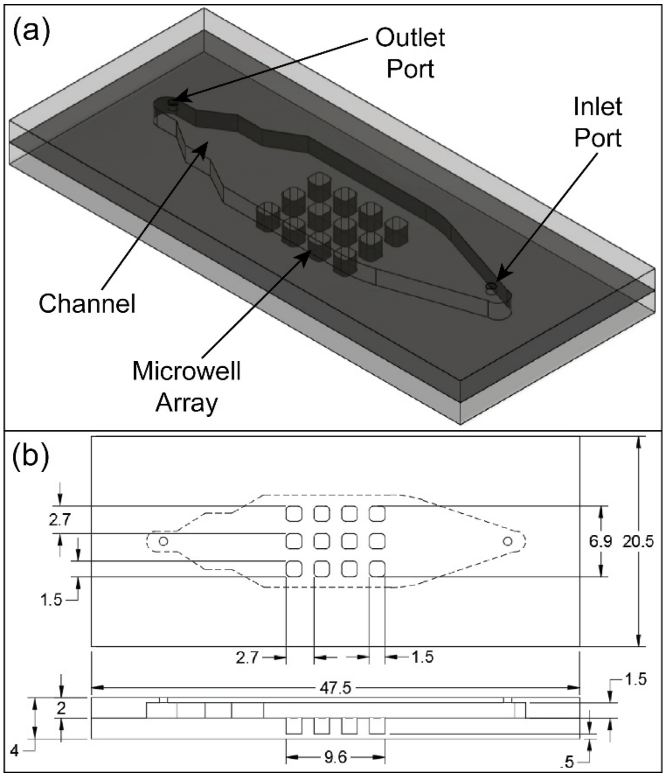


**Figure S3**: **Microwell chip design used for MAGI system evaluation.** (a) Isometric view of the 3x4 microwell array beneath a microfluidic channel for sample delivery and discretization. (b) Detailed device dimensions (units in mm).

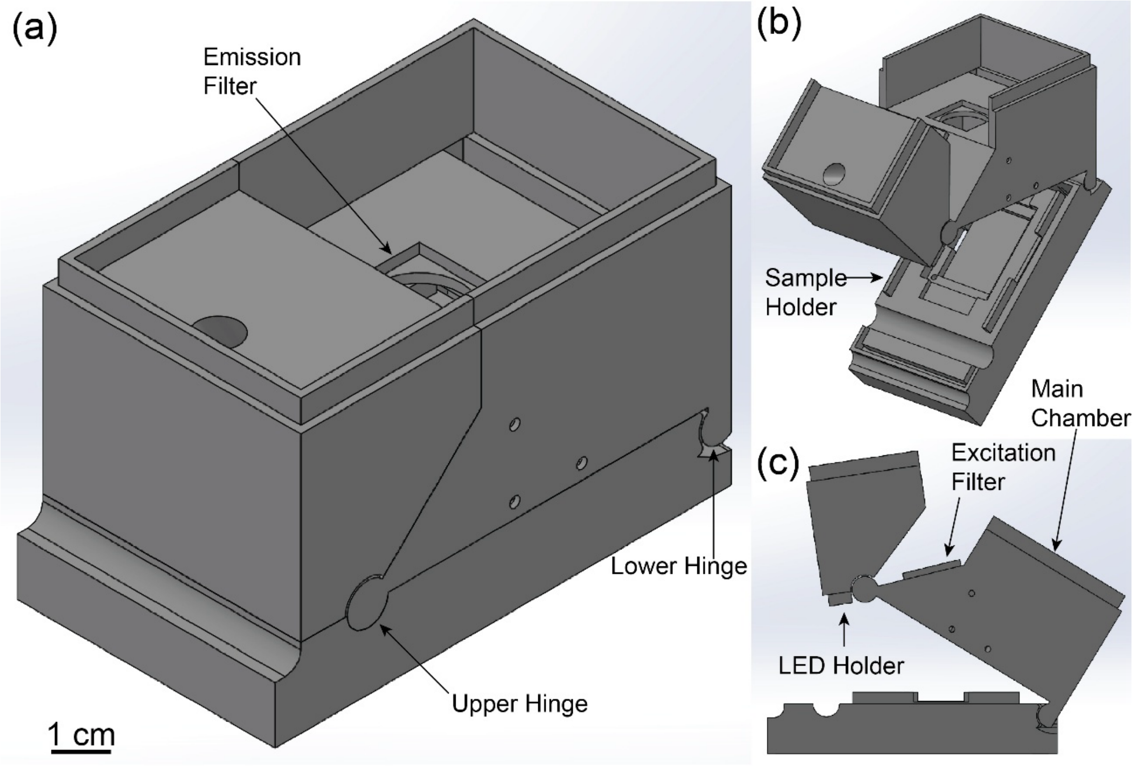


**Figure S4: MAGI case design.** (a) Details of the dual-hinged case design. The lower hinge provides access to the sample holder and the upper hinge provides access to the excitation LED during system assembly. (b) Isometric and (c) side views showing hinge operation.

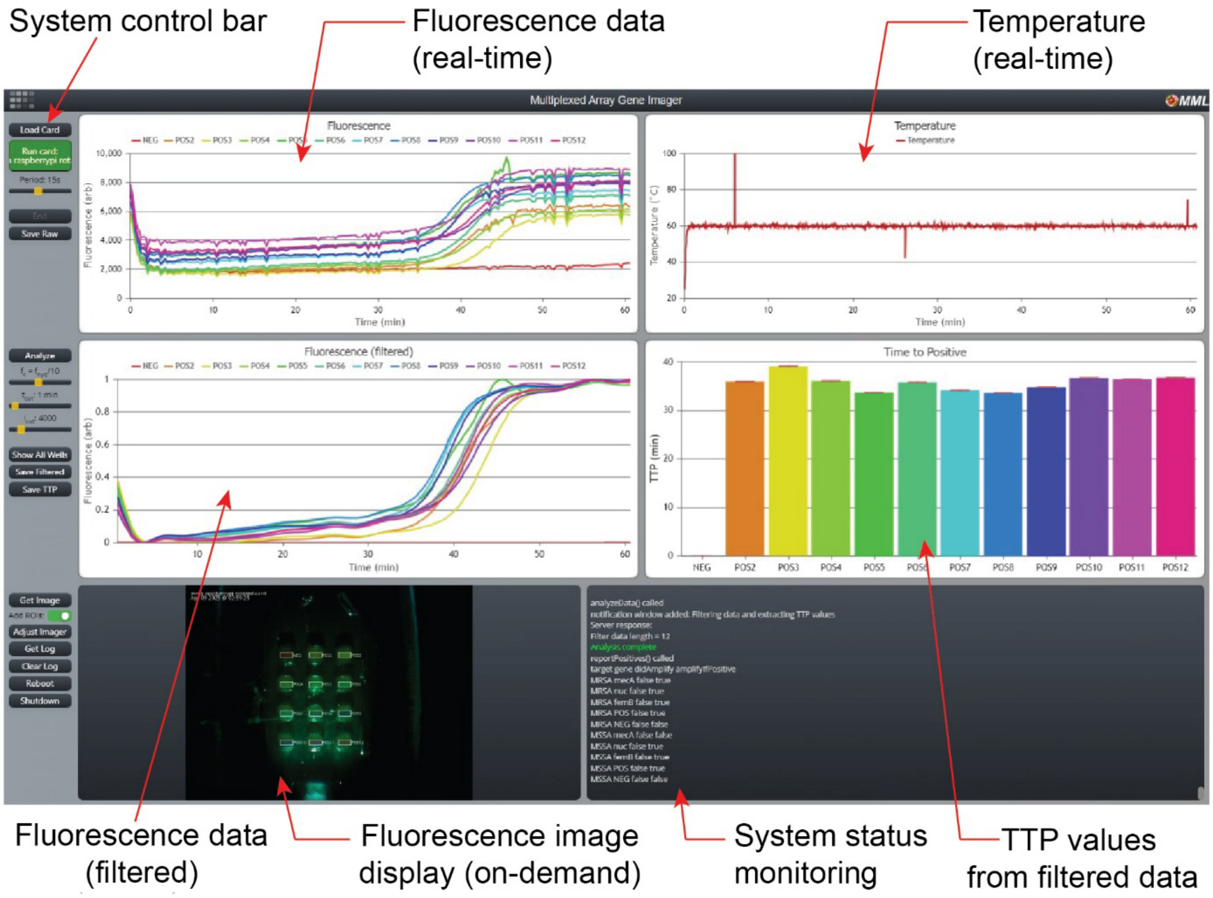


**Figure S5: Overview of the MAGI graphical interface.** The Javascript-based GUI provides a control bar for defining assay parameters, initiating or stopping an assay, and on-demand fluorescence image acquisition. Other key interface elements include real-time fluorescence measurements for each user-defined region of interest, real-time temperature data, and labeled fluorescence image data. A system status window provides user feedback on assay status.

**Table S1: Performance metrics for portable fluorescence-based nucleic acid amplification platforms.**

| | fluorescence detection? | Thermal control (cost) | integrated controller? | Flexible substrate options? | flexible target configuration? | area-based detection? | wireless or integrated operation? | volume (< 500 cm3?) |
|---|---|---|---|---|---|---|---|---|
| **Mendoza-Gallegos et al. 2018** | Y | ceramic resistor (low) | N | Y | Y | Y | N | 252* |
| **Jie et al. 2020** | Y | metal film on Si (high) | Y | N | N | Y | N | 216* |
| **Everitt et al. 2021** | Y | nichrome wire coil (low) | N | N | N | N | N | 225* |
| **Everitt et al. 2022** | Y | polyimide pad (high) | N | N | N | N | N | 166* |
| **Yu et al. 2022** | Y | metal ceramic block (high) | N | N | N | Y | N | 768* |
| **Trick et al. 2022** | Y | thermoelectric (high) | Y | N | N | N | integrated | 4541 |
| **Dong et al. 2023** | Y | metal ceramic block (high) | N | Y | Y | Y | N | 243* |
| **MAGI** | Y | copper on PCB (low) | Y | Y | Y | Y | wireless | 380 |

* estimated from published data

**Table S2: Transfer functions and prefilter parameters for the thermal control system.**

| | | |
|---|---|---|
| Overall controller | $T_d(s)$ | $\frac{11.6 \times 10^{-3}}{s^2 + 0.15s + 11.6 \times 10^{-3}}$ |
| Plant | $G(s)$ | $\frac{11.7 \times 10^{-3}}{s + 6.15 \times 10^{-3}}$ |
| PID controller | $G_c(s)$ | $\frac{12.38s + 0.99}{s}$ |
| Prefilter | $G_p(s)$ | $\frac{11.6 \times 10^{-3}}{0.14s + 11.6 \times 10^{-3}}$ |
| Discrete-time prefilter parameters | $a$ | 1.0 |
| | $b$ | $5.1 \times 10^{-5}$ |

**Table S3: MAGI system bill of materials.**

| Category | Description | Manufacturer | Part Number | Quantity | Cost | Total |
|---|---|---|---|---|---|---|
| Optics | Excitation Filter | Rosco | P1080 | **1 | $3.30 | $3.30 |
| | Emission Filter | Rosco | P5156 | **1 | $3.10 | $3.10 |
| | 5MP CMOS Camera | Okdo | 72480773 | 1 | $10.50 | $10.50 |
| Electronics | Raspberry Pi Zero 2 W with Header | Raspberry Pi | SC0721 | 1 | $18.00 | $18.00 |
| | 10-bit SAR ADC | Microchip Technology | MCP3008T-I/SL | 1 | *$2.20 | $2.20 |
| | Excitation LED | ams OSRAM | GV QSSPA1.13-JZKZ-V1V6-1 | 1 | *$0.72 | $0.72 |
| | Green LED | Cree LED | C4SMA-GGY-CU2W37A1 | 1 | *$0.11 | $0.11 |
| | Red LED | Cree LED | C4SMA-RGY-CT24QBB2 | 1 | *$0.11 | $0.11 |
| | Transistor | Infineon Technologies | IRFML8244TRPBF | 4 | *$0.14 | $0.56 |
| | 6.2 Ω Power Resistor | Bourns | PWR263S-20-6R20FE | 1 | $3.14 | $3.14 |
| | 220 Ω Resistor | Susumu | RR0816P-221-D | 2 | *$0.03 | $0.07 |
| | 2 kΩ Resistor | Panasonic | ERA-3AEB202V | 4 | *$0.07 | $0.28 |
| | 2 kΩ Thermistor | Measurement Specialties | GA2K7MCD1 | 2 | *$15.15 | $30.30 |
| | Fan | Nidec Components | F16FB-05LLC /E | 1 | *$5.99 | $5.99 |
| | 2x20 Female Header | Adafruit | 2243 | 1 | $1.95 | $1.95 |
| | System PCB | PCBWay | N/A | 1 | *$0.79 | $0.79 |
| | Heater PCB | PCBWay | N/A | 1 | $0.45 | $0.45 |
| | LED PCB | PCBWay | N/A | 1 | *$0.42 | $0.42 |
| | | | | | **Total** | **$81.99** |

*Assumed bulk purchasing of 100 units.
**Purchased a 2"x2" filter and cut it into 9 even squares.

**Note S1: Thermal control system design.**

Closed loop control over the sample temperature was implemented using a robust PID control scheme employing the integral of time multiplied by absolute error (ITAE) criterion (Ahamad et al. 2019; Simon et al. 2022; Zan et al. 2022). This method of control includes the reference temperature $R(s)$, a prefilter to condition the reference $G_p(s)$, a PID controller $G_c(s)$, the plant of the MAGI thermal system $G(s)$, and the output temperature $Y(s)$. A block diagram of the robust PID control loop is shown in **Fig. S3**. The plant transfer function $G(s)$ was assumed to be of the form of a first-order thermal system:

$$G(s) = \frac{\alpha * K}{s+\alpha} \quad (1)$$

To determine the transfer function, the steady state temperature was measured under different actuation duty cycles (4-24% at 4% increments), with noise filtered using a moving average filter with a window size of 20. The measured temperature response was plotted for each duty cycle, and curves were fitted of the form:

$$T(t) = (T_f - T_i)[1 - e^{-\alpha t}] \quad (2)$$

Values for $\alpha$ were extrapolated from all temperature response measurements and averaged for a final value. The measured steady-state temperature change, $T_f - T_i$, was also plotted against the duty cycle to find the gain ($K$) of the system from the slope of this plot.

Given target values for percent overshoot (%OS) and settling time ($T_s$), the closed loop natural frequency ($\omega_n$) was determined using Equations 3 and 4:

$$\zeta = \frac{-\log\left(\%OS/_{100}\right)}{\sqrt{\pi^2+\log^2\left(\%OS/_{100}\right)}} \quad (3)$$

$$\omega_n = \frac{4}{T_s * \zeta} \quad (4)$$

Using the optimum coefficients based on the second order ITAE Criterion for step inputs (Dorf 2011), the desired overall transfer function $T_d(s) = Y(s)/R(s)$ was then found as:

$$T_d(s) = \frac{\omega_n^2}{s^2+1.4\omega_n+\omega_n^2} \quad (5)$$

From the block diagram (**Fig. S2**) the overall system transfer function is given by

$$T_d(s) = G_p \frac{G_c G}{1+G_c G} \quad (6)$$

where the PID controller $G_c(s)$ has the form

$$G_c(s) = \frac{K_d s^2+K_i s+K_p}{s} \quad (7)$$

where $K_p$, $K_i$, and $K_d$ are the proportional, integral, and derivative coefficients, respectively. The PID coefficients may then be found by combining Eqn. 1 with Eqns. 5-7. Finally, once again combining Eqn. 1 with Eqns. 5-7, $G_p(s)$ was calculated as:

$$G_p(s) = \frac{\omega_n^2}{G_c G} \tag{8}$$

$G_p(s)$ ensured that the numerator of the overall controller matched the numerator of the desired transfer function. To apply $G_p(s)$ to the PID controller, the transfer function was converted into discrete-time form:

$$G_p(q) = \frac{b}{q-a} \tag{9}$$

This was achieved by substituting $s = (q\text{-}1)/t_s$ into $G_p(s)$. The sampling time $t_s$, i.e. the time between each PID update cycle, was found by averaging the difference between the times after each update. Once $G_p(q)$ was calculated, a and b were substituted into Eqn. 10. This equation takes the desired temperature, $r(k)$, and the current filtered reference, $r_F(k)$, to find $r_F(k+1)$, the next filtered reference. $r_F(k+1)$ was used as the setpoint in the PID controller, which was updated on every iteration of the control loop. While implementing $G_p$, the initial $r_F(k)$ was set to room temperature (23 °C).

$$r_F(k+1) = a r_F(k) + b r(k) \tag{10}$$

A MATLAB script was developed to find the PID and pre-filter parameters using $G(s)$, $t_s$, and the desired values of $T_s$ and %OS. The calculated transfer functions and pre-filter parameters are shown in **Table S2**.

**Note S2: Links to design file and code repositories.**

Electronic files required for MAGI device fabrication and operation are available through online repositories. The Open Science Framework repository at https://osf.io/8ck4b contains the following files:

1. SolidWorks and STL part files for modular case components
2. PCB layout files
3. Python script for measuring steady state temperature under varying duty cycle actuation
4. Python script for calculating the plant transfer function based on experimental steady state temperature data
5. Matlab script for control system tuning

All codes required to operate and interface with the MAGI system are available through a Github repository at https://github.com/mmlmems/magi.

**Note S3: Calculation of detection limit.**

A statistical approach was used to estimate the detection limit of the MAGI device by modeling the number of gDNA copies in each well using a Poisson distribution. This distribution is appropriate because the loading process represents a series of independent discrete events (capture of single gDNA molecules) with a mean number of copies per well that remains constant for a given sample concentration and well volume. The Poisson probability mass function is given by:

$$P(\lambda, k) = \frac{\lambda^k e^{-\lambda}}{k!} \tag{11}$$

where $k$ is the number of DNA molecules present in a given well, and λ is the expected number of copies per well defined by the product of the initial gDNA concentration ($C$) and volume of a single well ($V$):

$$\lambda = C \times V \tag{12}$$

The probability that a single well has more than $m$ molecules (i.e. is above the detection limit) is:

$$\begin{aligned} p = P(DNA > m) &= 1 - P(DNA \leq m) \\ &= 1 - \sum_{k=0}^{m} P(\lambda, k) = 1 - \sum_{k=0}^{m} \frac{\lambda^k e^{-\lambda}}{k!} \end{aligned} \tag{13}$$

Since detection occurs independently within each well, the expected number of positive wells ($p$) is defined in terms of the total number of wells ($N$) and the number of wells with positive amplification ($n$) as simply:

$$E[positive\ wells] = N \cdot p = n \tag{14}$$

Therefore:

$$p = \frac{n}{N} \tag{15}$$

Combining (13) and (15) yields:

$$1 - \sum_{k=0}^{m} \frac{\lambda^k e^{-\lambda}}{k!} = \frac{n}{N} \quad \longrightarrow \quad \sum_{k=0}^{m} \frac{\lambda^k e^{-\lambda}}{k!} = 1 - \frac{n}{N} \tag{16}$$

The estimated values for the detection limit ($m$) can thus be determined by evaluating the cumulative Poisson distribution function in (16). Stated equivalently, $m$ is given approximately by the $(1\text{-}n/N)^{th}$ quantile of the Poisson distribution. In our study using a total well count of $N$ = 12 with an individual well volume of $V$ = 3.375 μL, a positive LAMP reaction was observed in a single well ($n$ = 1) when using an initial sample concentration of $C = 1\times10^0$ cp/μL. Using these

values in equations (12) and (16), we find an estimated detection limit of $m = 6$ gDNA copies prior to amplification.

Uncertainty in the estimated detection limit arises from the fact the observed assay result reflects a random outcome using a limited number of wells. Because the number of positive wells follows a binomial distribution, the Wilson Score Interval can be used to determine an appropriate confidence interval for the detection limit. Using this approach, lower and upper bounds given on the probability that a single well contains more than $m$ molecules is given by:

$$p_{lower}, p_{upper} = \frac{p + \frac{z^2}{2N} \pm z\sqrt{\frac{p(1-p)}{N} + \frac{z^2}{4N^2}}}{1 + \frac{z^2}{N}} \tag{17}$$

where $p$ is the point estimate for the number of positive wells as given by equation (15), and $z = 1.96$ for a 95% confidence interval. The corresponding bounds on $m$ are then found from:

$$\sum_{k=0}^{m_{lower}} \frac{\lambda^k e^{-\lambda}}{k!} = 1 - p_{lower} \tag{18}$$

and:

$$\sum_{k=0}^{m_{upper}} \frac{\lambda^k e^{-\lambda}}{k!} = 1 - p_{upper} \tag{19}$$

Using this approach, we find the 95% confidence interval for the detection limit to be $m \in [3, 10]$ molecules, with a point estimate of 6 molecules. Dividing by $V = 3.375$ µL yields a corresponding 95% confidence interval for the concentration detection limit given by [0.9, 3.0] cp/µL, with a point estimate of:

$$\frac{6\ cp}{3.375\ \mu L} \approx 1.8\ {}^{cp}/_{\mu L} \tag{20}$$
